# The Impact of Health Literacy Status on the Comparative Validity and Sensitivity of an Interactive Multimedia Beverage Intake Questionnaire

**DOI:** 10.3390/nu9010005

**Published:** 2016-12-23

**Authors:** Lucy P. Hooper, Emily A. Myers, Jamie M. Zoellner, Brenda M. Davy, Valisa E. Hedrick

**Affiliations:** Department of Human Nutrition, Foods, and Exercise, Virginia Polytechnic Institute and State University, 295 West Campus Drive, Blacksburg, VA 24061, USA; lucyh92@vt.edu (L.P.H.); eamyers@vt.edu (E.A.M.); zoellner@vt.edu (J.M.Z.); bdavy@vt.edu (B.M.D.)

**Keywords:** technology, beverage consumption, sugar-sweetened beverages, dietary assessment methodology, health literacy, validity, sensitivity, responsiveness

## Abstract

Self-reported dietary assessment methods can be challenging to validate, and reporting errors for those with lower health literacy (HL) may be augmented. Interactive multimedia (IMM) based questionnaires could help overcome these limitations. The objectives of this investigation are to assess the comparative validity and sensitivity to change of an IMM beverage intake questionnaire (IMM-BEVQ) as compared to dietary recalls and determine the impact of HL. Adults completed three 24-h dietary recalls and the IMM-BEVQ at baseline and after a six-month intervention targeting either sugar-sweetened beverages (SSB) or physical activity. Correlations and paired-samples *t*-tests are presented. For validity (*n* = 273), intake of SSB (mean difference = 10.6 fl oz) and total beverage consumption (mean difference = 16.0 fl oz) were significantly different (*p* ≤ 0.001) at baseline between the IMM-BEVQ and dietary recalls for all participants. However, the differences in intake were generally greater in low HL participants than in adequate HL participants. For sensitivity (*n* = 162), change in SSB intake (mean difference = 7.2 fl oz) was significantly different (*p* ≤ 0.01) between pre-/post-IMM-BEVQ and pre-/post-dietary recalls, but not total beverage intake (mean difference = 7.6 fl oz) for all participants. Changes in SSB and total beverage intake were not significantly different for those with adequate HL. The IMM-BEVQ is a valid dietary assessment tool that is as responsive to detecting changes in beverage intake as dietary recalls. However, adults with lower HL may need additional guidance when completing the IMM-BEVQ.

## 1. Introduction

The various types of self-reported dietary assessment tools have prompted controversy regarding their ability to accurately and consistently capture an individual’s dietary intake [1]. Food intake records and dietary recalls have been recognized as the “gold standards” for valid, reliable, and non-invasive dietary assessment tools for community-based populations [1]. However, these methods are resource-intensive, highly burdensome to researchers and subjects, and are only able to provide recent dietary intake [1]. Thus, food frequency questionnaires (FFQ), which have less associated researcher and participant burden than food records/recalls, may be used as an alternative method for briefly and cost-effectively capturing an individual’s habitual dietary intake [1]. Despite the advantages gained with using FFQ, both FFQ and food records/recalls may prove to be challenging among individuals with limited literacy status [1,2]. In regards to food records/recalls, participants may alter their normal intake patterns, unintentionally forget items, or misinterpret portion sizes [1,3,4]. FFQ can be cognitively complex for lower literate audiences, often leading to unintentionally skipped questions and inaccurate estimations of frequency and portion size [5]. Generally, FFQ are self-administered paper-based questionnaires [6]; however, it may be difficult for researchers to manage and score multiple paper-administered FFQ and data within large epidemiological studies. These difficulties associated with paper-administered FFQ can result in the collection of incomplete and/or unreliable data [1,5,7].

Interactive multimedia (IMM) FFQ or audio computer-assisted self-interviews (ACASI) are being developed to address some of the difficulties associated with traditional paper-administered FFQ by improving the validity of self-reported data [7,8]. These automated FFQ operate by two-way communication through the means of visual aids, audio assistance, and direct data entry to circumvent incomplete or inaccurate data [1,7,8,9]. These interactive features are especially useful for improving accuracy in estimations of frequency and portion sizes, which are often difficult questions for FFQ respondents [5,10]. IMM FFQ can provide greater benefits than paper-administered FFQ due to lower subject burden, rapid data entry, and cost effective data collection and analysis [11,12]. Additionally, these computer-automated surveys could prove to be a useful assessment tool in clinical and community settings due to their instantaneous feedback and ability to decrease literacy barriers in order to better suit participants exhibiting low health literacy or other types of reading impairments [13,14].

Health literacy (HL) can be defined as the “degree to which individuals have the capacity to obtain, process, and understand basic health information and services needed to make appropriate health decisions” [15]. The widespread presence of low HL is a major concern because it has been associated with deficits in health knowledge, poor health outcomes, and increased health care costs [16,17,18]. The impact of participants’ literacy, socioeconomic status, and education level on FFQ validation has been analyzed in previous literature; however, the results among previous FFQ validation studies analyzing how these participant characteristics impact FFQ completion accuracy as compared to other validated dietary assessment tools (e.g., food records, 24-h recalls, and doubly labeled water) have been inconsistent. Some FFQ validation studies have found that low socioeconomic status, literacy, and/or education level can impair a participant’s ability to accurately complete a FFQ [19,20,21]; while others did not find these participant characteristics to have a significant impact on completion accuracy [22,23]. Thus, it is critical to assess how the utility of a FFQ is impacted by HL status.

The BEVQ-15 is a validated paper-administered FFQ developed to rapidly assess habitual beverage consumption in adults [24,25,26]. Specifically, the BEVQ-15 was developed to quantify the frequency and amounts of 15 beverage categories as well as sugar-sweetened beverage (SSB) consumption [25,26]. The BEVQ-15 has a Flesch-Kinkaid readability score of 4.8, equating to a 4th grade reading level [25]. However, the overall layout may be overly complex for an individual with lower HL, i.e., all 15 beverage categories are presented on a single page along with questions regarding how often and how much they consume of each beverage. An initial computer-administered version of the BEVQ-15 (IMM-BEVQ) was developed to address these issues, and a pilot study established preliminary comparative validity between this IMM version and the paper-administered version of the BEVQ-15 [9]. Based on initial findings, improvements were made to the IMM-BEVQ, and an updated version was made available [27].

Before a FFQ is deemed acceptable for use, it should demonstrate validity, reliability, and sensitivity to change [1,28]. Furthermore, the impact of a participant’s HL status on these factors should be evaluated. Thus, the purpose of the current investigation is to assess (1) the comparative validity between the IMM-BEVQ and the gold standard of dietary intake recalls; (2) the sensitivity to change of the IMM-BEVQ; and (3) the differences in validity and sensitivity to change between low and adequate HL participants.

## 2. Materials and Methods

### 2.1. Recruitment and Trial Details

Participants (*n* = 301) were recruited from medically-underserved rural regions (Medical Underservice Index score of 62 or less) in southwest Virginia for this trial, known as Talking Health [27,29]. Talking Health is a six-month, community-based, two-arm randomized controlled behavioral trial which targets SSB consumption behaviors among low socio-economic status adults, as compared with a matched-contact comparison group targeting physical activity behaviors. Recruitment details are published elsewhere; briefly, participants were recruited from April 2012 to June 2014 through various recruitment methods [27]. To be eligible, participants had to consume at least 200 kcal/day from SSB, as assessed by the BEVQ-15, prior to enrollment [9,24,25,26]. Additionally, participants had to be English-speaking adults ≥18 years old, report no physical activity limitations, and could not be currently enrolled in any other nutrition or physical activity programs. Participants were randomized into either the SSB intervention group (SIPsmartER) or the physical activity control group (MoveMore) after completing baseline assessments. The SIPsmartER group’s primary intervention goal was to reach the recommendation of less than 8 fl oz of SSB per day, and the MoveMore group’s primary intervention goal was to reach 150 min of moderate-intensity aerobic activity and muscle-strengthening activities on two or more days per week [30].

### 2.2. IMM-BEVQ

**Figure 1:**
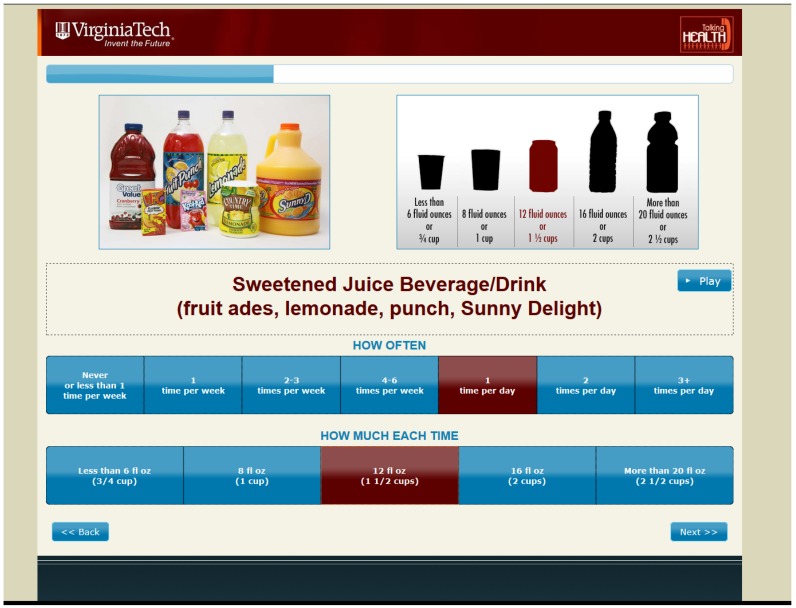
Screenshot example of the sweetened juice drink beverage category from the interactive multimedia beverage intake questionnaire (IMM-BEVQ-15).

The BEVQ-15 tool measures the amount and frequency consumed of 15 beverage categories including water, 100% fruit juice, sweetened juice drinks, whole milk, reduced fat milk, fat-free milk, regular soft drinks, diet soft drinks, sweet tea, sweetened coffee, black coffee/tea, beer, liquor, wine, and energy drinks [25,26]. Participants completed an in-person IMM-BEVQ at baseline and six-month data collection. The IMM-BEVQ provided several advantages over the traditional paper-administered BEVQ. Features associated with the IMM-BEVQ (Figure 1) included an audio option that read all instructions to the participant and read the selected answer option when the cursor hovered over it; all instructions could be repeated as well. The layout of the BEVQ-15 was expanded in order to present each beverage category on a separate page instead of presenting all of the beverages at one time/on the same page. For each beverage question, there were pictures and examples of the specific beverages as well as a portion size guide to help participants more accurately report their beverage consumption. After the answers for “how often” or “how much” were selected, the answer box turned maroon in color as well as the appropriate portion size diagram.

**Figure 2:**
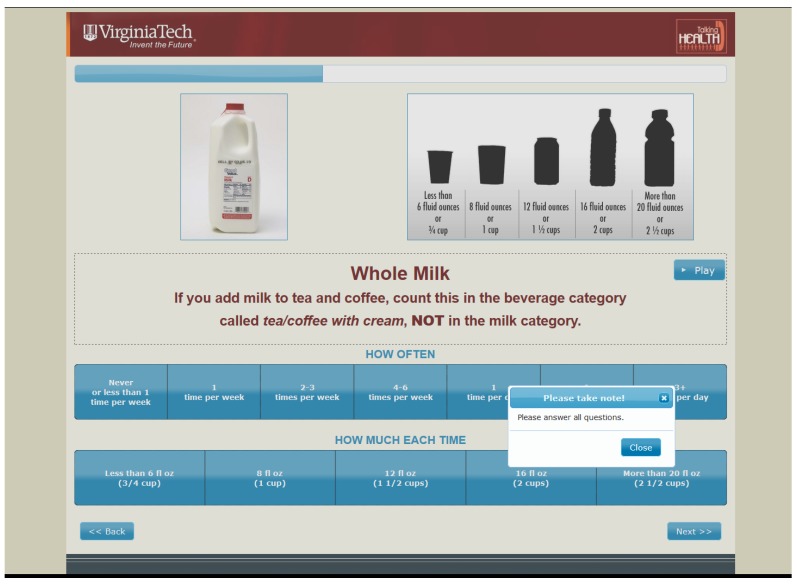
Screenshot example of attempting to move to the next beverage category without first answering the question from the interactive multimedia beverage intake questionnaire (IMM-BEVQ-15).

As seen in Figure 2, if participants inadvertently skipped a question, the computer would prompt them up to two times to answer the question before allowing them to move to the next beverage category. Finally, the IMM-BEVQ responses were automatically recorded and scored, which significantly reduced associated researcher-burden.

### 2.3. Dietary Recalls

Three dietary recalls were obtained at baseline data collection. The first 24-h dietary recall was completed during the in-person data collection session and on the same day as the IMM-BEVQ. The two remaining recalls were completed via unannounced telephone calls within a two-week period; the multiple pass method was used to collect the recalls, which were completed by trained research technicians who were supervised by a doctoral-level registered dietitian [31]. The dietary intake recalls were analyzed using the Nutrition Data System for Research (NDS-R) nutritional analysis software (Nutrition Coordinating Center, University of Minnesota, Minneapolis, MN, USA). Beverage consumption from the dietary recalls was extracted through the NDS-R food group output files.

### 2.4. Assessment of Health Literacy Status

The Newest Vital Sign is a valid and reliable HL assessment tool comprised of six questions based upon pertinent information displayed on nutrition facts labels [32,33]. All participants completed the interviewer-administered assessment at baseline to determine their HL status [34]. Participants were categorized with either low (0–3) or adequate (4–6) HL based on their score [34].

### 2.5. Demographics and Anthropometrics

Participants provided demographic information and underwent baseline and six-month follow up assessments of height, measured in meters without shoes using a portable stadiometer; weight, measured in light clothing without shoes, to the nearest 0.1 kg using a digital scale (model 310GS; Tanita, Tokyo, Japan); and calculated BMI.

### 2.6. Ethics

This study was conducted according to the guidelines laid down in the Declaration of Helsinki and the Virginia Tech Institutional Review Board approved the study protocol. Participants provided written informed consent prior to enrollment.

### 2.7. Statistical Analysis

**Figure 3:**
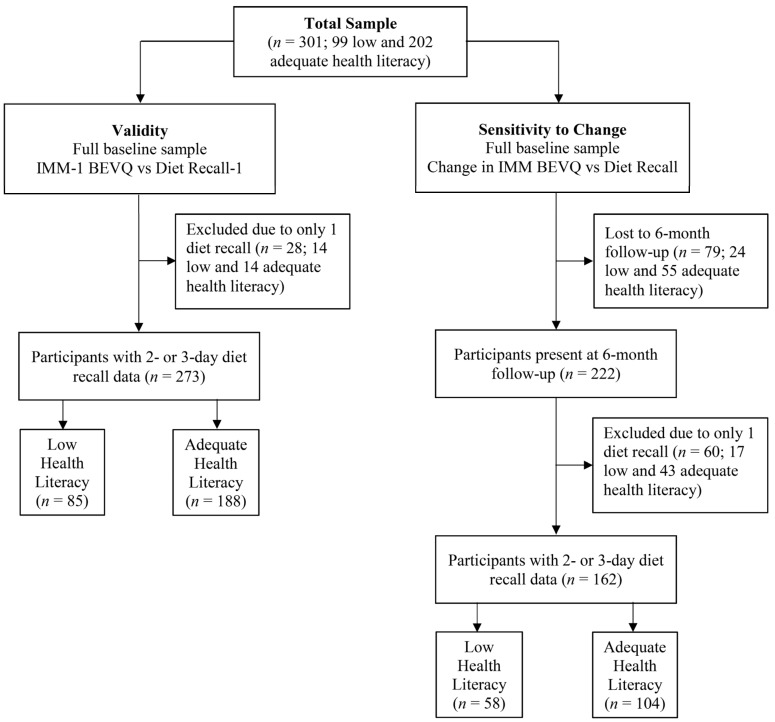
Flow Diagram of Analytical Sample Size.

Statistical analyses were performed using IBM SPSS statistical analysis software (v. 24 for Windows, 2016, SPSS Inc., Chicago, IL, USA). Descriptive statistics (mean ± standard deviation and frequencies) are reported for the participant demographic characteristics. One-way ANOVA and Chi-square analyses were utilized to assess for demographic differences between low and adequate HL participants. Figure 3 depicts a flow diagram of the sample sizes used for the validity and sensitivity analyses. The significance level was set a priori at *p* ≤ 0.05.

Validity (Aim 1) is the ability of a tool to accurately measure consumption of specific dietary items. For FFQ, this is typically accomplished through comparative validity in which FFQ responses are compared to intake from a “gold standard”, in this case, three 24-h dietary recalls. Thus, all participants’ baseline IMM-BEVQ responses were compared to baseline beverage consumption from the dietary recalls. Participants with only one complete dietary recall were excluded from this analysis, leaving an analytical sample of *n* = 273. Paired sample *t*-tests and bivariate correlations were used to compare the fluid ounces (fl oz) consumed within each beverage category as well as energy (kcal) and fl oz for total SSB and total beverage consumption. To further assess the similarity between the IMM-BEVQ and dietary recalls, Bland-Altman analyses were performed for total SSB and total beverages (fl oz and kcal). As participants were high SSB consumers, the data was slightly skewed; thus, log-transformed values were used for Bland-Altman analyses.

The ability of a tool to detect significant changes in dietary intake over time is known as sensitivity, or responsiveness, to change (Aim 2). The IMM-BEVQ’s sensitivity was assessed by comparing reported changes in the IMM-BEVQ to changes reported by a gold standard method (i.e., dietary recalls). Thus, both intervention arms were included in this analysis, as the magnitude of change was not an outcome, rather the magnitude of the difference between dietary assessment methods. For this investigation, changes in IMM-BEVQ responses over six-months were compared pre- and post-intervention (i.e., IMM-BEVQ-1 vs. IMM-BEVQ-2). To validate the changes reported by the IMM-BEVQ, the changes in beverage intake were compared to changes measured by the dietary recalls. Participants that did not return at the six-month data collection and those with only one complete dietary recall were excluded from this analysis, leaving an analytical sample of *n* = 162). Paired sample *t*-tests were used to compare the fl oz consumed within each beverage category as well as fl oz and kcal for total SSB and total beverage consumption.

For Aim 3, the previous methods for validity and sensitivity analyses were used for subsets of low and adequate HL participants.

## 3. Results

### 3.1. Demographic Characteristics

Within the analyses, one-third of participants were identified as low HL (Figure 3), and the majority of participants were Caucasian, female, and had a mean BMI in the obese category (Table 1). Within the validity sample, significant differences (*p* ≤ 0.001) in educational attainment and income were found between low and adequate HL participants. Twice as many adequate HL participants had some college or greater (80%) as compared to low HL participants (44%), and mean income for those with adequate HL was significantly greater than those with low HL ($26,649 ± 17,630 vs. $17,441 ± 14,790, respectively). The same significant demographic differences (*p* ≤ 0.001) were found for the sensitivity sample.

### 3.2. Comparative Validity

#### 3.2.1. All Participants

**Figure 4:**
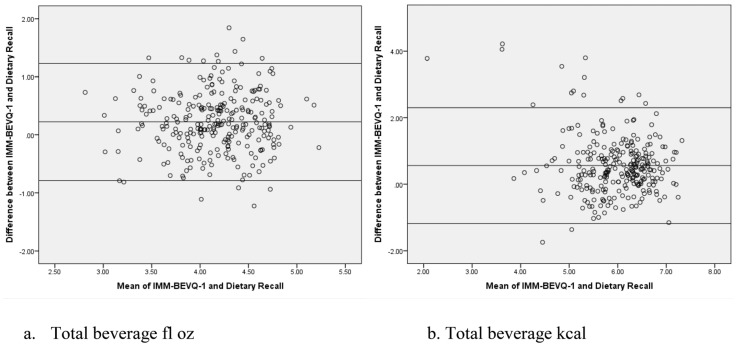

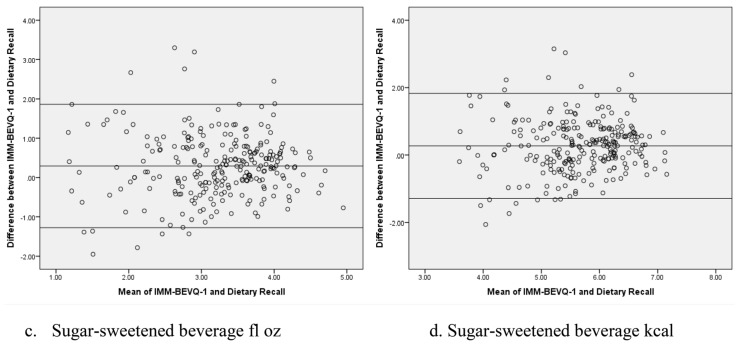
Bland-Altman analysis of IMM-BEVQ-1 and Dietary Recalls for (**a**) total beverage fl oz; (**b**) total beverage kcal; (**c**) total sugar-sweetened beverage fl oz; and (**d**) total sugar-sweetened beverage kcal. All values are log-transformed. The center line represents the mean difference and the upper and lower lines indicate the mean ± 1.96 standard deviation.

When examining all participants’ baseline data, there were no significant differences between the IMM-BEVQ-1 and dietary recalls for black coffee/tea, beer, and liquor (Table 2). All the other beverage categories were found to be significantly different, with differences between the IMM-BEVQ-1 and dietary recalls ranging from 0.1 to 3.3 fl oz on individual beverage categories. Additionally, reported intake of total SSB (mean differences = 10.6 fl oz and 124 kcal) and total beverage consumption (mean differences = 16.0 fl oz and 268 kcal) were significantly different between the IMM-BEVQ-1 and dietary recall. For all beverages with significant differences (with the exception of water), intake reflected by the IMM-BEVQ-1 was greater than intake reported on the dietary recalls. In addition, Bland-Altman analyses revealed a good agreement between the IMM-BEVQ-1 and the dietary recalls for total SSB fl oz and kcal (96% and 94%, respectively) as well as total beverage fl oz and kcal (both 94%) (Figure 4).

Intake across all beverage categories was significantly correlated (*p* ≤ 0.01) between the IMM-BEVQ-1 and dietary recalls (*r* ranged from 0.17 to 0.78), with the exception of sweetened juice drinks. Additionally, total SSB fl oz and kcal (*r* = 0.46 and 0.49, respectively) and total beverage consumption fl oz and kcal (*r* = 0.35 for both) were significantly correlated (*p* ≤ 0.001).

#### 3.2.2. Low versus Adequate Health Literate Participants

When the participants were dichotomized into low and adequate HL groups, the significant differences above persisted for most of the beverage categories. For both low and adequate HL groups, water and sweetened coffee were no longer significantly different. Additionally, for the low HL group, fat-free milk and wine were no longer significantly different, and black tea/coffee became significantly different. For the adequate HL group, the significant differences in sweetened coffee and energy drinks did not persist.

However, the significant difference in intake was generally greater in the low HL participants than in the adequate HL participants, especially regarding total SSB (mean difference between low and adequate HL participants = 8.3 fl oz and 105 kcal) and total beverage consumption (mean difference between low and adequate HL participants = 7.6 fl oz and 163 kcal). Bland-Altman analysis for the low and adequate HL groups was similar to findings from the total sample, however, the adequate HL participants did demonstrate slightly greater agreement (94%–97%) when compared to the low HL participants (92%–95%). Furthermore, low HL participants had fewer significantly correlated beverage categories, including 100% fruit juice and energy drinks (r ranged from 0.24 to 0.86; *p* ≤ 0.05). Low HL participants also typically demonstrated lower correlations, with the exception of black coffee, artificially sweetened drinks, beer, and wine, as compared to adequate HL participants, which demonstrated significant correlations for all beverage categories (*r* ranged 0.20 to 0.74; *p* ≤ 0.01), except sweetened juice drinks.

Total SSB and total beverage consumption between the IMM-BEVQ-1 and dietary recalls were significantly correlated for both HL groups. However, the correlation values were greater for adequate HL participants (*r* ranged 0.41 to 0.57; *p* ≤ 0.001) as compared to low HL participants (*r* ranged 0.27 to 0.39; *p* ≤ 0.05).

### 3.3. Sensitivity to Change

#### 3.3.1. All Participants

For the IMM-BEVQ, over the six-month intervention, intake of water significantly increased by 5.2 fl oz and total SSB consumption significantly decreased (mean decrease = 18.1 fl oz and 212 kcal), including significant decreases in regular soft drinks, sweet tea, and sweetened coffee (Table 3). A significant decrease in total beverage intake (mean decrease = 11.8 fl oz and 234 kcal) was also demonstrated. These results were verified by pre- and post-beverage intake reported by dietary recalls, with the exception of sweetened coffee and total beverage fl oz, which were not significantly different.

Importantly, minimal significant differences between changes in the IMM-BEVQ versus changes in the dietary recalls were found, including sweetened juice drinks, regular soft drinks, and sweet tea, with these differences ranging from 1.7 to 4 fl oz. Additionally, reported changes in intake of total SSB (mean differences = 7.2 fl oz and 92 kcal) and total beverage consumption kcal (mean difference = 102 kcal) were significantly different between pre-/post-IMM-BEVQ and pre-/post-dietary recall, but not total beverage fl oz (mean difference = 7.6 fl oz).

#### 3.3.2. Low versus Adequate Health Literate Participants

For the IMM-BEVQ over the six-month intervention, the differences above persisted for low HL participants with the exception of water, sweetened coffee, and total beverage fl oz no longer being significantly different and energy drinks became significantly different. The adequate HL participants showed significant differences within the same beverage categories as the total sample, with the exception of significantly different whole and reduced fat milk categories. These results were corroborated by pre- and post-beverage intake reported by dietary recalls, with the exception of water and sweet tea, which were not significantly different, and wine, which was significantly different.

Importantly, only one significant difference, for sweet tea (mean difference = 3.1 fl oz), between changes in the IMM-BEVQ versus changes in the dietary recalls was found for the adequate HL group. Additionally, the adequate HL group reported changes in intake of total SSB (mean differences = 4 fl oz and 52 kcal) and total beverage consumption fl oz and kcal (mean differences = 6 fl oz and 74 kcal) which were not significantly different between pre-/post-IMM-BEVQ and pre-/post-dietary recall.

## 4. Discussion

### 4.1. Comparative Validity

Although the full sample revealed several significant (yet minimal) differences in individual beverage categories between the IMM-BEVQ-1 and dietary recalls, individuals with low HL generally demonstrated greater differences as compared to adequate HL individuals. Differences in total SSB and total beverage kcal were larger than differences reported from similar studies (mean difference of 8–44 kcal for SSB and 15–63 kcal for total beverages) [9,24,25]. Even so, correlations for the total sample were consistent with acceptable correlation ranges from other validation studies, which ranged from 0.4 to 0.7 according to the National Cancer Institute’s Register for Validated Short Dietary Assessment Instruments [35,36], as well as several individual investigations examing the associations between dietary recalls and FFQ outcomes [37,38,39,40]; however, the lower HL group fell slightly below this range at 0.3–0.4. These findings regarding differences in HL status are consistent with previous work validating an initial pilot IMM-BEVQ version against the paper/pencil version [9]. There are several possible explanations for the significant differences in beverage categories. The first is the platform of the IMM-BEVQ. Participants may feel they can be more honest reporting their consumption on a self-administered IMM or ACASI questionnaire [8,13] versus dietary recalls, where they may experience fear of judgment regarding their intake [33]. This may be especially prevalent when specifically examining consumption of socially undesirable items such as SSB [41]. In this sample, reported consumption of individual and total SSB was significantly greater on the IMM-BEVQ versus the dietary recalls; however, there were no significant differences in healthier beverages, such as water. Thus, reported intake on the IMM-BEVQ may be more representative of actual beverage consumption. Another reason for significant differences between the IMM-BEVQ and beverage intake from dietary recalls is the period of time that intake is measured. The BEVQ-15 is designed to measure habitual beverage intake over the past month, while dietary recalls are only able to measure recent beverage intake over three days. For example, if someone consumes soda three times per week, there is potential that the three dietary recalls would not include any soda consumption, while the BEVQ-15 would be able to capture this intake. Finally, it has been demonstrated that HL status plays a substantial role in one’s ability to accurately determine portion sizes [5,35]. This may be one explanation for the significant differences in responses between the IMM-BEVQ and the dietary recalls as the IMM-BEVQ has pre-determined portion sizes for individuals to select, while individuals must determine appropriate portion sizes on the dietary recalls without prompts.

### 4.2. Sensitivity to Change

In line with the primary focus of the SSB reduction intervention, it was expected that the IMM-BEVQ would detect significant decreases in SSB consumption and significant increases in low energy density beverages over the six-month intervention for SIPsmartER participants [30]. Furthermore, it was hypothesized that exposure to beverage related questions throughout the intervention and during data collection [30], as well as increased physical activity [42], could have influenced MoveMore participant’s beverage intake behaviors during the six-month period. Accordingly, the results exhibited significantly decreased consumption of individual and total SSB (e.g., regular soft drinks, sweet tea, sweetened coffee) and significantly increased consumption of low energy density beverages (e.g., water). When comparing mean differences between responses for the IMM-BEVQ and dietary recalls over the six-month period, total SSB fl oz and kcal and total beverage kcal were significantly different; however, the significant differences did not persist when examining adequate HL participants. Proposed reasons for these differences are similar to those posed in the validity discussion above. Despite it being suggested that the sensitivity, or responsiveness, of a dietary assessment tool is just as important as validity and reproducibility, sensitivity is not considered or studied extensively in the literature [43,44]. Without sensitive and rapid measures of dietary consumption, extensive longitudinal data must be collected, and consequently, it is challenging to overcome weaknesses in the current body of literature surrounding beverage consumption. Thus, the availability of an IMM-BEVQ-15 would facilitate the ability to rapidly measure and immediately obtain information regarding beverage consumption patterns, as compared to a paper and pencil version of the BEVQ-15. This would be useful for researchers to measure changes in beverage intake in response to dietary interventions, for practitioners to track patients’ intake and provide targeted feedback to individuals desiring to improve their dietary habits, or for individuals to track their beverage consumption behaviors over time.

### 4.3. Strengths & Limitations

In order to evaluate the comparative validity and sensitivity to change of a dietary assessment questionnaire, a large sample size (*n* = 223 and *n* = 162) and comparison to a “gold standard” dietary assessment method for community-based populations (e.g., two to three day dietary recalls) were used in this study [27,33,45]. Evaluation and classification of HL status within each of the analyses provided additional value to the IMM-BEVQ by assessing its comparative validity and sensitivity among low HL populations, which are at an increased risk of excessive SSB consumption and consequently, related co-morbidities [30].

When validating self-report dietary assessment tools, limitations are often present and must be acknowledged. First, within this study, participant diversity was lacking in terms of race/ethnicity and gender, with the majority of participants being Caucasian and female; however, the participant demographics, with the exception of gender, were representative of U.S. census data for the region [46]. The rural region of southwest Virginia was targeted as it contains a higher proportion of low socio-economic status adults, nonetheless, the recruited sample was diverse in education and income as low socio-economic status was not required for acceptance into the study. Furthermore, participants had to consume at least 200 kcal per day of SSB to be eligible, which may have contributed to a higher percentage of obese individuals as compared to national rates [27]. In order to generalize these results among all HL levels in future studies, low HL individuals may require additional assistance in estimating portion sizes, following directions, and classifying beverages into appropriate beverage categories, such as juice and milk beverages. It is also critical to consider participant bias and reporting errors that could have occurred in the self-reported assessments utilized in this study [1]. To help offset the potential bias, gold-standard dietary recall methodology and state-of the-art nutritional analysis (NDS-R) software was used. Additionally, PhD-level registered dietitians oversaw all dietary data collection.

## 5. Conclusions

This investigation determined that the IMM-BEVQ demonstrates acceptable comparative validity and it is as responsive to detecting changes in adults’ habitual beverage intake patterns as dietary intake recalls. However, adults with lower health literacy status may need additional guidance when completing the IMM-BEVQ (i.e., not self-administered, provide assistance with categorizing beverages, and physical portion size models). Nonetheless, in comparison to a paper-administered questionnaire, the IMM version has several advantages such as two-dimensional portion size models, audio feature that reads instructions aloud, additional beverage examples with pictures, and ability to alert participants to skipped questions. The IMM-BEVQ is a rapid and cost-effective assessment tool that could provide an alternative method for researchers and practitioners to detect changes in beverage consumption among those participating in dietary interventions.

## Figures and Tables

**Table 1:** Participant Characteristics by Analytical Sample.

Characteristics	Validity Sample (*n* = 273) *n* (%)	Sensitivity Sample (*n* = 162) *n* (%)
Sex		
Male	46 (17)	29 (18)
Female	227 (83)	133 (82)
Mean Age (years) ± SD	42.0 ± 13.4	43.3 ± 13.3
Race/Ethnicity		
White	256 (94)	153 (94.5)
African American	12 (4)	7 (4.5)
Other	5 (2)	2 (1)
Mean BMI (kg/m^2^) ± SD	33.1 ± 9.1	33.3 ± 9.2
BMI Categories		
Underweight (≤18.4)	4 (2)	2 (1)
Normal weight (18.5–24.9)	54 (20)	30 (18.5)
Overweight (25–29.9)	61 (22)	35 (21.5)
Obese (≥30)	154 (56)	95 (59)
Education Level		
<High school	29 (10.5)	17 (10.5)
High school graduate	56 (20.5)	35 (21.5)
≥Some college	188 (69)	110 (68)
Annual Household Income ($)		
Mean income ± SD	23,782 ± 17,306	24,907 ± 18,466
≤14,999	113 (41.5)	68 (42)
15,000–34,999	88 (32)	44 (27)
35,000–54,999	37 (13.5)	22 (14)
≥55,000	35 (13)	28 (17)

**Table 2:** Comparative validity of an interactive multimedia beverage intake questionnaire (IMM-BEVQ): Comparison to dietary recalls at baseline data collection.

Beverage Category	Health Literacy Level	IMM-BEVQ-1 ^a^	Dietary Recall ^a^	Mean Difference ^b^
Water fl oz	Total (*n* = 273)	21.3 (18.7)	24.1 (25.6)	2.8 (21.6) *
	Low (*n* = 85)	20.6 (19.5)	23.2 (24.9)	2.6 (23.8)
	Adequate (*n* = 188)	21.6 (18.3)	24.5 (26.0)	2.9 (20.7)
100% Fruit Juice fl oz	Total	2.9 (4.5)	0.7 (1.9)	2.3 (4.6) ***
	Low	3.5 (5.7)	0.5 (1.5)	3.0 (5.6) ***
	Adequate	2.7 (3.9)	0.8 (2.0)	1.9 (4.0) ***
Sweetened Juice Drinks fl oz	Total	3.7 (8.9)	1.0 (2.5)	2.6 (9.1) ***
	Low	4.9 (10.1)	0.9 (2.4)	4.0 (10.4) **
	Adequate	3.1 (8.3)	1.1 (2.6)	2.0 (8.4) **
Whole Milk fl oz	Total	3.3 (9.0)	0.5 (1.8)	2.8 (8.5) ***
	Low	4.7 (11.4)	0.4 (1.1)	4.3 (11.2) **
	Adequate	2.7 (7.7)	0.6 (2.0)	2.1 (6.9) ***
Reduced Fat Milk fl oz	Total	2.6 (5.1)	1.1 (2.9)	1.5 (4.7) ***
	Low	2.3 (4.6)	1.1 (2.3)	1.2 (4.5) *
	Adequate	2.7 (5.3)	1.1 (3.1)	1.6 (4.8) ***
Fat-Free Milk fl oz	Total	1.6 (4.5)	0.9 (3.1)	0.7 (3.9) **
	Low	0.5 (1.7)	0.3 (1.5)	0.2 (1.9)
	Adequate	2.1 (5.3)	1.1 (3.5)	1.0 (4.5) **
Regular Soft Drinks fl oz	Total	14.8 (17.9)	12.2 (15.6)	2.6 (13.4) **
	Low	16.5 (18.5)	12.9 (16.4)	3.6 (15.8) *
	Adequate	14.0 (17.6)	11.8 (15.2)	2.1 (12.1) *
Artificially Sweetened Drinks fl oz	Total	6.4 (13.6)	4.1 (11.0)	2.3 (8.5) ***
	Low	6.6 (14.2)	4.4 (10.8)	2.2 (7.4) **
	Adequate	6.3 (13.3)	3.9 (11.1)	2.4 (9.0) ***
Sweet Tea fl oz	Total	9.0 (13.9)	5.7 (11.7)	3.3 (12.7) ***
	Low	8.5 (14.6)	3.7 (7.5)	4.9 (14.5) **
	Adequate	9.2 (13.6)	6.6 (13.0)	2.6 (11.8) **
Sweetened Coffee fl oz	Total	8.9 (12.5)	7.0 (17.1)	1.8 (14.1) *
	Low	6.6 (11.1)	4.4 (8.4)	2.2 (10.2)
	Adequate	9.9 (13.0)	8.3 (19.8)	1.7 (15.5)
Black Coffee/Tea fl oz	Total	2.7 (8.8)	4.1 (11.1)	1.3 (11.2)
	Low	2.6 (8.1)	5.4 (13.5)	2.9 (12.2) *
	Adequate	2.8 (9.2)	3.4 (9.9)	0.6 (10.6)
Beer fl oz	Total	1.3 (5.2)	0.9 (4.8)	0.4 (4.5)
	Low	1.8 (7.5)	1.4 (6.7)	0.4 (6.4)
	Adequate	1.0 (3.7)	0.7 (3.6)	0.3 (3.4)
Liquor fl oz	Total	0.1 (0.6)	0.1 (1.3)	0.0 (1.3)
	Low	0.1 (0.5)	0.0 (0.0)	0.1 (0.5)
	Adequate	0.1 (0.6)	0.2 (1.6)	0.0 (1.6)
Wine fl oz	Total	0.2 (0.7)	0.1 (0.6)	0.1 (0.8) *
	Low	0.1 (0.5)	0.1 (0.4)	0.1 (0.6)
	Adequate	0.3 (0.8)	0.1 (0.6)	0.1 (0.9) *
Energy Drinks fl oz	Total	2.3 (6.3)	1.3 (4.0)	1.0 (6.4) *
	Low	3.8 (9.5)	0.8 (3.0)	3.0 (9.3) **
	Adequate	1.6 (3.9)	1.5 (4.3)	0.1 (4.2)
Total Sugar-Sweetened Beverages fl oz	Total	38.2 (28.8)	27.6 (23.4)	10.6 (27.4) ***
	Low	39.9 (34.6)	23.6 (19.6)	16.3 (32.5) ***
	Adequate	37.5 (25.9)	29.5 (24.7)	8.0 (24.5) ***
Total Sugar-Sweetened Beverage kcal	Total	440 (351)	316 (254)	124 (317) ***
	Low	477 (421)	281 (242)	196 (395) ***
	Adequate	422 (314)	331 (259)	91 (269) ***
Total Beverage fl oz	Total	80.3 (39.9)	64.3 (31.4)	16.0 (41.2) ***
	Low	81.9 (47.9)	60.8 (31.8)	21.2 (49.7) ***
	Adequate	79.5 (35.9)	65.9 (31.2)	13.6 (36.6) ***
Total Beverage kcal	Total	659 (478)	391 (279)	268 (460) ***
	Low	723 (586)	344 (252)	380 (567) ***
	Adequate	630 (420)	412 (288)	217 (395) ***

^a^ Reported values are means (standard deviation); ^b^ Reported values are absolute mean differences (standard deviation) at baseline between IMM-BEVQ-1 and dietary recalls via paired samples *t*-tests; slight differences may be noted from the preceding columns due to rounding; * *p* ≤ 0.05; ** *p* ≤ 0.01; *** *p* ≤ 0.001.

**Table 3:** Sensitivity to change of an interactive multimedia beverage intake questionnaire (IMM-BEVQ) over a six-month intervention targeting either beverage consumption or physical activity.

Beverage Category	Health Literacy Level	IMM-BEVQ-1 ^a^	IMM-BEVQ-2 ^a^	Mean Difference between IMM 1 and 2 ^b^	Mean Difference between Dietary Recall 1 and 2 ^c^	Mean Difference between IMM Change and Recall Change ^d^
Water fl oz	Total (*n* = 162)	21.2 (18.7)	26.4 (19.0)	5.2 (20.5) **	5.5 (26.6) **	0.4 (29.9)
	Low (*n* = 58)	20.4 (18.0)	26.3 (20.5)	5.9 (23.0)	7.0 (30.4)	1.0 (39.3)
	Adequate (*n* = 104)	21.6 (19.2)	26.4 (18.3)	4.7 (19.2) *	4.6 (24.3)	0.0 (23.5)
100% Fruit Juice fl oz	Total	3.2 (5.0)	2.7 (6.6)	0.5 (7.1)	0.0 (2.7)	0.5 (7.5)
	Low	4.3 (6.5)	3.5 (9.6)	0.8 (11.2)	0.1 (1.6)	0.6 (11.2)
	Adequate	2.6 (3.9)	2.2 (4.2)	0.4 (3.4)	0.1 (3.1)	0.5 (4.4)
Sweetened Juice Drinks fl oz	Total	3.9 (9.4)	2.2 (7.8)	1.8 (11.5)	0.0 (4.6)	1.7 (11.2) *
	Low	6.1 (11.8)	3.4 (11.1)	2.7 (16.4)	0.1 (5.0)	2.6 (15.3)
	Adequate	2.8 (7.6)	1.5 (4.9)	1.3 (7.5)	0.0 (4.5)	1.2 (8.0)
Whole Milk fl oz	Total	3.0 (8.6)	1.9 (5.4)	1.1 (7.8)	0.3 (2.2)	0.8 (7.4)
	Low	3.7 (9.7)	3.1 (7.2)	0.6 (10.1)	0.0 (1.7)	0.7 (10.0)
	Adequate	2.7 (8.0)	1.3 (3.9)	1.4 (6.2) *	0.5 (2.4) *	0.9 (5.5)
Reduced Fat Milk fl oz	Total	2.5 (4.9)	2.3 (5.0)	0.2 (5.4)	0.3 (3.4)	0.0 (6.2)
	Low	3.0 (5.4)	3.8 (7.0)	0.7 (8.1)	0.2 (3.3)	0.7 (8.8)
	Adequate	2.2 (4.7)	1.5 (3.2)	0.7 (3.0) *	0.3 (3.5)	0.4 (4.1)
Fat-Free Milk fl oz	Total	1.6 (4.2)	2.8 (9.0)	1.2 (8.5)	0.2 (5.6)	1.0 (10.3)
	Low	0.5 (1.6)	2.2 (8.5)	1.6 (8.6)	0.1 (1.6)	1.7 (8.4)
	Adequate	2.2 (5.1)	3.2 (9.3)	1.0 (8.5)	0.3 (6.9)	0.7 (11.2)
Regular Soft Drinks fl oz	Total	14.3 (17. 2)	6.8 (12.7)	7.5 (16.0) ***	4.8 (12.7) ***	2.8 (15.3) *
	Low	15.8 (18.4)	5.5 (10.3)	10.3 (16.8) ***	5.9 (14.6) **	4.8 (14.8) *
	Adequate	13.5 (16.5)	7.6 (13.8)	6.0 (15.5) ***	4.2 (11.6) ***	1.7 (15.5)
Artificially Sweetened Drinks fl oz	Total	6.3 (13.5)	7.1 (12.8)	0.8 (13.4)	0.2 (11.8)	0.5 (14.4)
	Low	7.6 (14.9)	6.6 (13.5)	1.0 (2.0)	0.3 (10.7)	0.4 (11.8)
	Adequate	5.7 (12.6)	7.5 (12.5)	1.8 (12.4)	0.5 (12.4)	1.0 (15.6)
Sweet Tea fl oz	Total	9.1 (14.8)	4.1 (9.5)	5.1 (14.2) ***	1.1 (10.0) **	4.0 (14.9) **
	Low	8.3 (15.2)	3.4 (9.6)	4.9 (16.6) *	0.5 (9.3)	5.5 (15.5) *
	Adequate	9.6 (14.7)	4.5 (9.4)	5.1 (12.8) ***	2.0 (10.2)	3.1 (14.6) *
Sweetened Coffee fl oz	Total	9.5 (14.1)	6.5 (10.8)	3.0 (11.3) **	4.3 (18.1)	1.3 (18.8)
	Low	6.3 (12.1)	5.7 (9.7)	0.7 (12.6)	1.6 (9.2)	0.7 (15.1)
	Adequate	11.2 (14.8)	6.9 (11.4)	4.3 (10.4) ***	5.8 (21.4) **	1.6 (20.5)
Black Coffee/Tea fl oz	Total	3.2 (9.5)	3.8 (9.1)	0.6 (11.5)	1.4 (14.4)	0.9 (17.0)
	Low	3.0 (9.2)	4.6 (11.3)	1.5 (12.7)	0.9 (13.4)	2.5 (17.6)
	Adequate	3.3 (9.7)	3.4 (7.6)	0.1 (10.8)	2.8 (14.8)	2.8 (16.5)
Beer fl oz	Total	1.6 (6.7)	1.7 (6.8)	0.1 (6.7)	0.1 (6.3)	0.2 (6.1)
	Low	2.1 (8.9)	2.8 (9.9)	0.7 (10.2)	0.7 (9.4)	0.0 (8.7)
	Adequate	1.4 (4.9)	1.1 (3.9)	0.3 (3.2)	0.5 (3.6)	0.2 (3.9)
Liquor fl oz	Total	0.1 (0.5)	0.1 (0.4)	0.0 (0.3)	0.0 (0.1)	0.0 (0.3)
	Low	0.1 (0.6)	0.1 (0.5)	0.0 (0.4)	0.0 (0.0)	0.0 (0.4)
	Adequate	0.1 (0.4)	0.1 (0.4)	0.0 (0.2)	0.0 (0.1)	0.0 (0.3)
Wine fl oz	Total	0.2 (0.7)	0.2 (0.8)	0.0 (0.7)	0.1 (0.9)	0.0 (1.0)
	Low	0.2 (0.5)	0.1 (0.8)	0.1 (0.8)	0.1 (1.2)	0.1 (0.7)
	Adequate	0.3 (0.8)	0.2 (0.8)	0.1 (0.7)	0.2 (0.7)*	0.1 (1.1)
Energy Drinks fl oz	Total	1.8 (3.7)	1.1 (5.1)	0.6 (5.9)	0.4 (3.9)	0.4 (5.3)
	Low	2.3 (4.1)	0.9 (2.1)	1.4 (3.3) **	0.4 (3.5)	1.4 (3.4) **
	Adequate	1.5 (3.4)	1.3 (6.3)	0.2 (6.9)	0.4 (4.2)	0.2 (6.1)
Total Sugar Sweetened Beverages fl oz	Total	38.4 (30.8)	20.3 (23.6)	18.1 (31.8) ***	10.8 (23.7) ***	7.2 (33.8) **
	Low	38.5 (37.0)	18.2 (23.9)	20.3 (40.0) ***	7.3 (19.4) **	13.0 (36.4) **
	Adequate	38.4 (26.9)	21.5 (23.5)	16.9 (26.2) ***	12.7 (25.6) ***	4.0 (32.0)
Total Sugar-Sweetened Beverages kcal	Total	438 (369)	227 (286)	212 (382) ***	118 (249) ***	92 (377) *
	Low	462 (446)	208 (299)	254 (478) ***	91 (236) **	163 (427) **
	Adequate	426 (319)	239 (279)	187 (316) ***	134 (256) ***	52 (341)
Total Beverage fl oz	Total	80.8 (43.3)	69.0 (40.4)	11.8 (45.5) **	4.3 (30.9)	7.6 (53.5)
	Low	82.0 (54.1)	70.3 (52.1)	11.7 (62.0)	1.1 (34.1)	10.6 (70.0)
	Adequate	80.1 (36.2)	68.2 (32.2)	11.9 (33.2) ***	6.0 (29.0) *	6.0 (41.8)
Total Beverage kcal	Total	655 (510)	422 (487)	234 (506) ***	131 (280) ***	102 (506) *
	Low	715 (623)	474 (652)	243 (677) **	89 (258) *	154 (649)
	Adequate	621 (435)	393 (365)	228 (383) ***	154 (291) ***	74 (407)

^a^ Reported values are means (standard deviation); ^b^ Reported values are absolute mean differences (standard deviation) between IMM-BEVQ-1 (baseline) and IMM-BEVQ-2 (six-month follow-up) via paired samples *t*-tests; slight differences may be noted from the preceding columns due to rounding; ^c^ Reported values are absolute mean differences (standard deviation) between baseline dietary recalls (1) and six-month dietary recalls (2) via paired samples *t*-tests; ^d^ Reported values are absolute mean differences (standard deviation) between baseline to six-month changes in IMM-BEVQ responses (IMM-BEVQ-1 and IMM-BEVQ-2) and baseline to six-month changes in dietary recall responses (dietary recall 1 and dietary recall 2) via paired samples *t*-tests; slight differences may be noted from the preceding columns due to rounding; * *p* ≤ 0.05; ** *p* ≤ 0.01; *** *p* ≤ 0.001.

## References

[1] Thompson F.E., Subar A.F., Boushey C.J., Coulston A.M., Rock C.L., Monsen E.R. (2001). Dietary Assessment Methodology. Nutrition in the Prevention and Treatment of Disease.

[2] Johnson R., Soultanakis R., Matthews D. (1998). Literacy and Body Fatness are Associated with Underreporting of Energy Intake in US Low-Income Women Using the Multiple-Pass 24-h Recall: A Doubly Labeled Water Study. J. Am. Diet. Assoc..

[3] Smith A.F. (1993). Cognitive psychological issues of relevance to the validity of dietary reports. Eur. J. Clin. Nutr..

[4] Jonnalagadda S.S., Mitchell D.C., Smiciklas-Wright H., Meaker K.B., van Heel N., Karmally W., Ershow A.G., Kris-Etherton P.M. (2000). Accuracy of energy intake data estimated by a multiple-pass, 24-h dietary recall technique. J. Am. Diet. Assoc..

[5] Huizinga M.M., Carlisle A.J., Cavanaugh K.L., Davis D.L., Gregory R.P., Schlundt D.G., Rothman R.L. (2009). Literacy, numeracy, and portion-size estimation skills. Am. J. Prev. Med..

[6] Falomir Z., Arregui M., Madueño F., Corella D., Coltell Ó. (2012). Automation of Food Questionnaires in Medical Studies: A state-of-the-art review and future prospects. Comput. Biol. Med..

[7] Kristal A.R., Kolar A.S., Fisher J.L., Plascak J.J., Stumbo P.J., Weiss R., Paskett E.D. (2014). Evaluation of web-based, self-administered, graphical food frequency questionnaire. J. Acad. Nutr. Diet..

[8] Brown J.L., Swartzendruber A., DiClemente R.J. (2013). Application of Audio Computer-Assisted Self-Interviews to Collect Self-Reported Health Data: An Overview. Caries Res..

[9] Riebl S.K., Paone A.C., Hedrick V.E., Zoellner J.M., Estabrooks P.A., Davy B.M. (2013). The comparative validity of interactive multimedia questionnaires to paper-administered questionnaires for beverage intake and physical activity: Pilot study. JMIR Res. Protoc..

[10] Smith A.F., Jobe J.B., Mingay D.J. (1991). Question-induced cognitive biases in reports of dietary intake by college men and women. Health Psychol..

[11] Greenlaw C., Brown-Welty S. (2009). A comparison of web-based and paper-based survey methods: Testing assumptions of survey mode and response cost. Eval. Rev..

[12] Ekman A., Dickman P.W., Klint A., Weiderpass E., Litton J.-E. (2006). Feasibility of using web-based questionnaires in large population-based epidemiological studies. Eur. J. Epidemiol..

[13] Jantz C., Anderson J., Gould S.M. (2002). Using computer-based assessments to evaluate interactive multimedia nutrition education among low-income predominantly Hispanic participants. J. Nutr. Educ. Behav..

[14] Long J.D., Littlefield L.A., Estep G., Martin H., Rogers T.J., Boswell C., Shriver B.J., Roman-Shriver C.R. (2010). Evidence review of technology and dietary assessment. Worldviews Evid. Based. Nurs..

[15] Ratzan S.C., Parker R.M., Selden C., Zorn M., Ratzan S.C., Parker R.M. (2000). Introduction. National Library of Medicine Current Bibliographies in Medicine: Health Literacy.

[16] Yin H.S., Johnson M., Mendelsohn A.L., Abrams M.A., Sanders L.M., Dreyer B.P. (2009). The health literacy of parents in the United States: A nationally representative study. Pediatrics.

[17] Schillinger D., Grumbach K., Piette J., Wang F., Osmond D., Daher C., Palacios J., Sullivan G.D., Bindman A.B. (2002). Association of health literacy with diabetes outcomes. JAMA.

[18] Parker R.M., Ratzan S.C., Lurie N. (2003). Health literacy: A policy challenge for advancing high-quality health care. Health Aff..

[19] Kristal A.R., Feng Z., Coates R.J., Oberman A., George V. (1997). Associations of race/ethnicity, education, and dietary intervention with the validity and reliability of a food frequency questionnaire: The Women’s Health Trial Feasibility Study in Minority Populations. Am. J. Epidemiol..

[20] Marks G.C., Hughes M.C., van der Pols J.C. (2006). Relative validity of food intake estimates using a food frequency questionnaire is associated with sex, age, and other personal characteristics. J. Nutr..

[21] Tooze J.A., Vitolins M.Z., Smith S.L., Arcury T.A., Davis C.C., Bell R.A., DeVellis R.F., Quandt S.A. (2007). High levels of low energy reporting on 24-h recalls and three questionnaires in an elderly low-socioeconomic status population. J. Nutr..

[22] Johansson G., Wikman Å., Åhrén A.-M., Hallmans G., Johansson I. (2001). Underreporting of energy intake in repeated 24-h recalls related to gender, age, weight status, day of interview, educational level, reported food intake, smoking habits and area of living. Public Health Nutr..

[23] Heitmann B.L. (1993). The influence of fatness, weight change, slimming history and other lifestyle variables on diet reporting in Danish men and women aged 35–65 years. Int. J. Obes. Relat. Metab. Disord..

[24] Hedrick V.E., Comber D.L., Estabrooks P.A., Savla J., Davy B.M. (2010). The beverage intake questionnaire: Determining initial validity and reliability. J. Am. Diet. Assoc..

[25] Hedrick V.E., Savla J., Comber D.L., Flack K.D., Estabrooks P.A., Nsiah-Kumi P.A., Ortmeier S., Davy B.M. (2012). Development of a brief questionnaire to assess habitual beverage intake (BEVQ-15): Sugar-sweetened beverages and total beverage energy intake. J. Acad. Nutr. Diet..

[26] Hedrick V.E., Comber D.L., Ferguson K.E., Estabrooks P.A., Savla J., Dietrich A.M., Serrano E., Davy B.M. (2013). A rapid beverage intake questionnaire can detect changes in beverage intake. Eat. Behav..

[27] Zoellner J., Chen Y., Davy B., You W., Hedrick V., Corsi T., Estabrooks P. (2014). Talking health, a pragmatic randomized-controlled health literacy trial targeting sugar-sweetened beverage consumption among adults: Rationale, design & methods. Contemp. Clin. Trials.

[28] Cade J.E., Burley V.J., Warm D.L., Thompson R.L., Margetts B.M. (2004). Food-frequency questionnaires: A review of their design, validation and utilisation. Nutr. Res. Rev..

[29] Medically Underserved Areas/Populations: Guidelines for MUA and MUP Designation. https://bhw.hrsa.gov/shortage-designation/muap.

[30] Zoellner J.M., Hedrick V.E., You W., Chen Y., Davy B.M., Porter K.J., Bailey A., Lane H., Alexander R., Estabrooks P.A. (2016). Effects of a behavioral and health literacy intervention to reduce sugar-sweetened beverages: A randomized-controlled trial. Int. J. Behav. Nutr. Phys. Act..

[31] Stote K.S., Radecki S.V., Moshfegh A.J., Ingwersen L.A., Baer D.J. (2011). The number of 24 h dietary recalls using the US Department of Agriculture’s automated multiple-pass method required to estimate nutrient intake in overweight and obese adults. Public Health Nutr..

[32] Zoellner J., You W., Connell C., Smith-Ray R.L., Allen K., Tucker K.L., Davy B.M., Estabrooks P. (2011). Health literacy is associated with healthy eating index scores and sugar-sweetened beverage intake: Findings from the rural Lower Mississippi Delta. J. Am. Diet. Assoc..

[33] Willett W. (1998). Nutritional Epidemiology.

[34] Weiss B.D., Mays M.Z., Martz W., Castro K.M., DeWalt D.A., Pignone M.P., Mockbee J., Hale F.A. (2005). Quick assessment of literacy in primary care: The newest vital sign. Ann. Fam. Med..

[35] Thompson F.E., Subar A.F. (2013). Dietary Assessment Methodology. Nutrition in the Prevention and Treatment of Disease.

[36] National Cancer Instititue’s Register of Validated Short Dietary Assessment Instruments. https://epi.grants.cancer.gov/diet/shortreg/.

[37] KrisEtherton P., Eissenstat B., Jaax S., Srinath U., Scott L., Rader J., Pearson T. (2001). Validation for MEDFICTS, a Dietary Assessment Instrument for Evaluating Adherence to Total and Saturated Fat Recommendations of the National Cholesterol Education Program Step 1 and Step 2 Diets. J. Am. Diet. Assoc..

[38] Buscemi S., Rosafio G., Vasto S., Massenti F.M., Grosso G., Galvano F., Rini N., Barile A.M., Maniaci V., Cosentino L. (2015). Validation of a food frequency questionnaire for use in Italian adults living in Sicily. Int. J. Food Sci. Nutr..

[39] Birgisdottir B.E., Kiely M., Martinez J.A., Thorsdottir I. (2008). Validity of a food frequency questionnaire to assess intake of seafood in adults in three European countries. Food Control.

[40] Andersen L., Johansson L., Solvoll K. (2002). Usefulness of a short food frequency questionnaire for screening of low intake of fruit and vegetable and for intake of fat. Eur. J. Public Health.

[41] Kuhnle G.G. (2012). Nutritional biomarkers for objective dietary assessment. J. Sci. Food Agric..

[42] Halliday T.M., Davy B.M., Clark A.G., Baugh M.E., Hedrick V.E., Marinik E.L., Flack K.D., Savla J., Winett S., Winett R.A. (2014). Dietary intake modification in response to a participation in a resistance training program for sedentary older adults with prediabetes: Findings from the Resist Diabetes study. Eat. Behav..

[43] Kristal A.R., Beresford S.A., Lazovich D. (1994). Assessing change in diet-intervention research. Am. J. Clin. Nutr..

[44] Guyatt G., Walter S., Norman G. (1987). Measuring change over time: Assessing the usefulness of evaluative instruments. J. Chronic Dis..

[45] Monsen E.R. (2003). Research: Successful Approaches.

[46] US Census Bureau (2010) American FactFinder Fact Sheet: Lee County, Giles County, Pulaski County, Washington County, Grayson County, Wise County, VA, USA. http://factfinder2.census.gov.

